# Anus-Preserving Surgery in Advanced Low-Lying Rectal Cancer: A Perspective on Oncological Safety of Intersphincteric Resection

**DOI:** 10.3390/cancers13194793

**Published:** 2021-09-24

**Authors:** Guglielmo Niccolò Piozzi, Se-Jin Baek, Jung-Myun Kwak, Jin Kim, Seon Hahn Kim

**Affiliations:** Division of Colon and Rectal Surgery, Department of Surgery, Korea University Anam Hospital, Korea University College of Medicine, Seoul 02841, Korea; guglielmopiozzi@gmail.com (G.N.P.); xezin@korea.ac.kr (S.-J.B.); jmkwak@korea.ac.kr (J.-M.K.); mrgs@korea.ac.kr (J.K.)

**Keywords:** advanced rectal cancer, intersphincteric resection, minimally invasive surgery, anus-preserving surgery, sphincter-saving surgery, robotic surgery, laparoscopic surgery, pelvic local recurrence, abdominoperineal resection, anatomy

## Abstract

**Simple Summary:**

Intersphincteric resection (ISR) is the ultimate anus-preserving surgical technique for very low-lying rectal cancers. The oncological safety of ISR has been frequently discussed, especially relatively to abdominoperineal resection. This review critically discusses the oncological safety of ISR by evaluating the anatomical characteristics of the deep pelvis, the clinical indications, the role of distal and circumferential resection margins, the role of the neoadjuvant chemoradiotherapy, the outcomes between surgical approaches (open, laparoscopic, and robotic), the comparison with abdominoperineal resection, the risk factors for oncological outcomes and local recurrence, the patterns of local recurrences after ISR, considerations on functional outcomes after ISR, and learning curve and surgical education on ISR.

**Abstract:**

The surgical management of low-lying rectal cancer, within 5 cm from the anal verge (AV), is challenging due to the possibility, or not, to preserve the anus with its sphincter muscles maintaining oncological safety. The standardization of total mesorectal excision, the adoption of neoadjuvant chemoradiotherapy, the implementation of rectal magnetic resonance imaging, and the evolution of mechanical staplers have increased the rate of anus-preserving surgeries. Moreover, extensive anatomy and physiology studies have increased the understanding of the complexity of the deep pelvis. Intersphincteric resection (ISR) was introduced nearly three decades ago as the ultimate anus-preserving surgery. The definition and indication of ISR have changed over time. The adoption of the robotic platform provides excellent perioperative results with no differences in oncological outcomes. Pushing the boundaries of anus-preserving surgeries has risen doubts on oncological safety in order to preserve function. This review critically discusses the oncological safety of ISR by evaluating the anatomical characteristics of the deep pelvis, the clinical indications, the role of distal and circumferential resection margins, the role of the neoadjuvant chemoradiotherapy, the outcomes between surgical approaches (open, laparoscopic, and robotic), the comparison with abdominoperineal resection, the risk factors for oncological outcomes and local recurrence, the patterns of local recurrences after ISR, considerations on functional outcomes after ISR, and learning curve and surgical education on ISR.

## 1. Introduction

The surgical management of low-lying rectal cancer (LRC), defined as tumors located within 4–5 cm from the anal verge (AV) or 2 cm above the dentate line (DL), is technically and oncologically challenging. The difficult aim is to obtain an oncologically safe resection within the narrow bony boundaries of the pelvis by sparing the anus with its functioning sphincter complex, optimizing the post-surgical quality of life. This is possible today thanks to several improvements in surgical techniques. Abdominoperineal resection (APR), with a permanent colostomy, has long been the standard of care for rectal cancer [1,2], however, it is now limited mainly to LRC [3]. Anterior resection [4] and its technical evolution, with the development of mechanical staplers allowing a double stapling technique [5], was the first effective anus-preserving technique for rectal cancer [6]. Total mesorectal excision (TME) [7] turned the tide for surgical treatment of rectal cancer. Heald et al. reported an oncological improvement between conventional surgery plus radiotherapy and TME alone with a reduction of the 5 years local recurrence (LR) rate (25 to 5%, respectively) and 5 years overall recurrence rate (62.7 to 22%, respectively) for Dukes stage B2 and C [8,9]. The adoption of a multimodal treatment for rectal cancer with the development of neoadjuvant chemoradiotherapy (nCRT) protocols has furtherly improved the LR rate allowing tumor down-staging with up to 20% of complete response (depending on the waiting period) and possible improvement in sphincter preservation rate [10,11,12]. Moreover, the precise staging with rectal magnetic resonance imaging (MRI) has widely changed the approach to all rectal cancers [13,14]. All these advancements together have reduced the LR rate from above 50% to below 10% [15,16,17].

Intersphincteric resection (ISR), followed by hand-sewn coloanal anastomosis (CAA), is the ultimate anus-preserving technique [18]. ISR allows larger distal resection margins for LRC, providing acceptable oncological and functional outcomes, and avoiding a permanent stoma [18,19].

This review discusses the oncological safety of ISR by presenting the following aspects: (1) anatomy of the deep pelvis; (2) definition of ISR; (3) indications of ISR; (4) distal resection margin; (5) circumferential resection margin; (6) nCRT; (7) surgical approach (open, laparoscopic, robotic); (8) risk factors for oncological outcomes; (9) comparison with APR; (10) patterns of LR after ISR; (11) considerations on functional outcomes after ISR; (12) learning curve and surgical education on ISR.

## 2. Anatomy of the Deep Pelvis

Thorough knowledge of the deep pelvic anatomy is the first essential step for oncologically curative rectal surgery. The deep pelvis is characterized by a complex relationship between the rectum/anal canal, the genitourinary complex, and the pelvic floor. The anatomic complexity is not only on a macroscopic level but also, and principally, on a microscopic level. Both should be known by modern rectal surgeons to perform oncologically safe and curative resections. The adoption of minimally invasive approaches and the consequences of camera magnification has increased the visible details of the pelvis, especially at the anorectal junction, increasing the surgeon’s interest in microscopic anatomy.

The introduction of ISR has encouraged the need to recognize and define precise and clear landmarks for a safe oncological dissection. Modern anatomical studies, through the combined use of immunohistochemistry, MRI, and endoscopic ultrasound (EUS) both on cadaveric and live patients, have been performed in the deep pelvis, with a surgeon’s point of view.

Three different regions (posterior, anterior, and lateral) characterize the deep pelvis, each with specific anatomical landmarks for a correct oncologically safe dissection of the intersphincteric plane (ISP) [20,21].

### 2.1. Posterior Anatomy

The rectal wall consists of five layers, from the lumen outward: mucosa (columnar epithelium); deep mucosa (lamina propria and muscularis mucosae); submucosa; muscularis propria with the inner-circular (CM) and outer-longitudinal muscle (LM); and serosa (perirectal fat, absent in the extraperitoneal rectum). 

**Figure 1:**
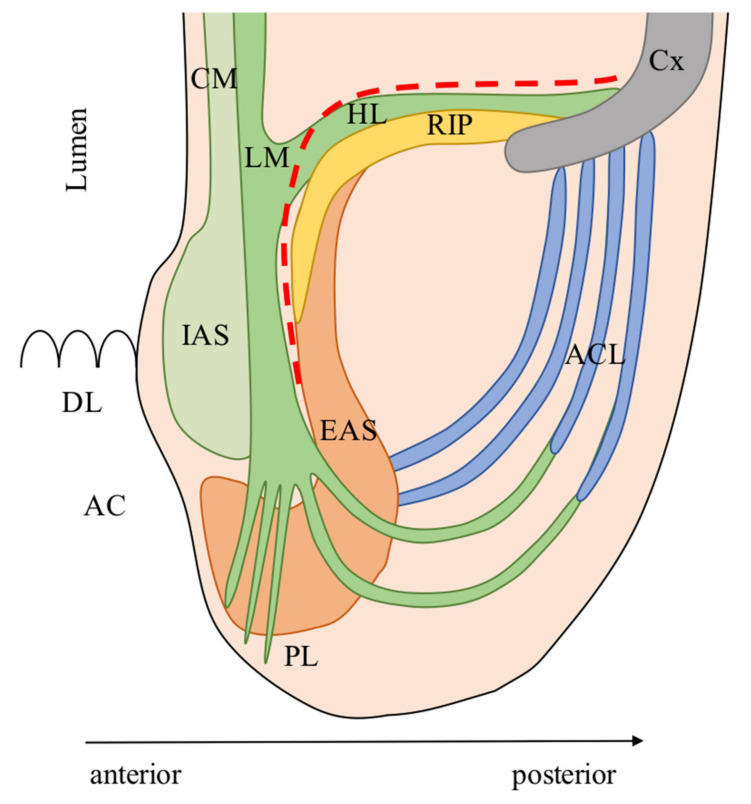
Posterior anatomy. AC: Anal canal; ACL: Anococcygeal ligament; Cx: Coccyx; CM: Circular muscle of the anal canal; EAS: External anal sphincter; HL: Hiatal ligament (sometimes referred as anococcygeal ligament by colorectal surgeons); IAS: Internal anal sphincter; LM: Longitudinal muscle of the anal canal; PL: Parks’ ligament; RIP: Raphe of iliococcygeus and pubococcygeus muscle; Red line: Dissection plane during intersphincteric resection. The anatomic model was designed according to the descriptions of Muro et al. [22]. Modified and reprinted with permission from ref. [20]. Copyright 2020 Turkish Journal of Colorectal Disease.

The smooth muscle cells of the muscularis propria continue caudally in the anal canal at the anorectal junction (Figure 1). The CM becomes the internal anal sphincter (IAS), which is coated externally by the caudal extension of the LM.

The smooth muscles cells of the LM have a complex spatial distribution [23]. Some fibers detach and run posteriorly to cover the skeletal muscle fibers of the levator ani muscle (LAM); these form the hiatal ligament (HL) [24]. The HL lays above all the surface of the LAM and continues anteriorly in the rectourethralis muscle (RU) as a single smooth muscle structure (described in detail in the anterior anatomy paragraph).

Caudally, the LM penetrates the inferior part (i.e., subcutaneous) of the external anal sphincter (EAS) splitting into fibers running anteriorly and inferiorly and terminating subcutaneously (Parks’ ligament or corrugator cutis ani muscle, PL) [25] and others running posteriorly and cranially forming a ligamentous loop composed of collagenous and elastic fibers terminating on the dorsal side of the coccyx. Muro et al. have defined this ligamentous loop as the anococcygeal ligament (ACL) following Toldt’s description in 1903 in an attempt to standardize the international anatomic terms [22,26]. The ACL separates two posterior spaces: deep and superficial post-anal space [27].

The EAS is located circumferentially and externally to the LM and has been historically described to be composed of three sheets (deep, superficial, and subcutaneous) [28], however recent studies describe it as a single continuous structure [29]. EAS is a skeletal muscle that continues cranially into the LAM, which is traditionally described as composed of three portions (puborectalis, pubococcygeus, and iliococcygeus). During surgery, the EAS and the LAM appear as macroscopically continuous muscular structures due to their tight anatomic connections [29]. The LAM adheres tightly to the ventral surface of the coccyx through a dense connective tissue called raphe of the iliococcygeus and pubococcygeus muscle (RIP) [22]. The thick tissue located above the LAM (HL) is composed of smooth muscle cells and connects the posterior aspect of the LM to the ventral surface of the coccyx [22,30]. This was named by Muro et al. as the hiatal ligament (HL) according to a previous description from Shafik [22,31], however, it is sometimes traditionally classified as anococcygeal ligament by colorectal surgeons. The ISP is located between the outer surface of the LM and the inner surface of the EAS. The HL is the surgical landmark for the posterior region of the deep pelvis and has to be dissected close to the posterior aspect of the rectum to access the posterior ISP [21]. Care must be taken not to perforate the rectum anteriorly or run through the RIP, into the deep post-anal space, posteriorly.

### 2.2. Anterior Anatomy

This region is characterized by a tight relationship between the genitourinary and anorectal complex. Lately, few anatomists and surgeons have examined this region with great precision through cadaveric dissections and anorectal EUS [29,32,33,34]. Thorough anatomical knowledge is crucial to optimize ISP identification and to obtain an oncologically safe dissection, avoiding any injuries. 

**Figure 2:**
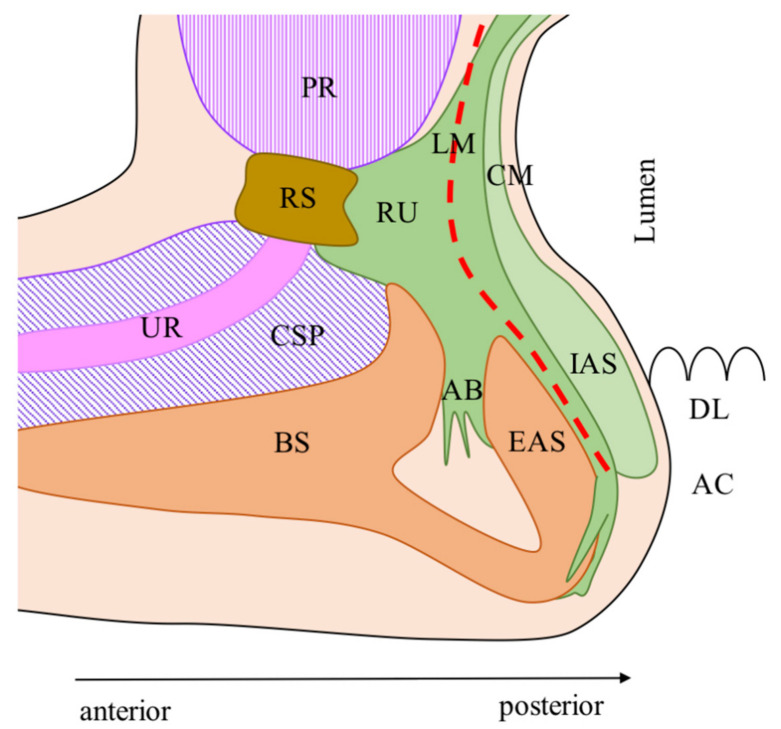
Male’s anterior anatomy. AB: Anterior bundle of the LM; AC: Anal canal; BS: Bulbospongiosus muscle; CM: Circular muscle of the anal canal; CSP: Corpus spongiosum of the penis; DL: Dentate line; EAS: External anal sphincter; IAS: Internal anal sphincter; LM: Longitudinal muscle of the anal canal; PR: Prostate; RS: Urethral rhabdosphincter; RU: Rectourethralis muscle; UR: Urethra; Red line: Dissection plane during intersphincteric resection. The anatomic model was designed according to the descriptions of Nakajima et al. [32]. Modified and reprinted with permission from ref. [20]. Copyright 2020 Turkish Journal of Colorectal Disease.

**Figure 3:**
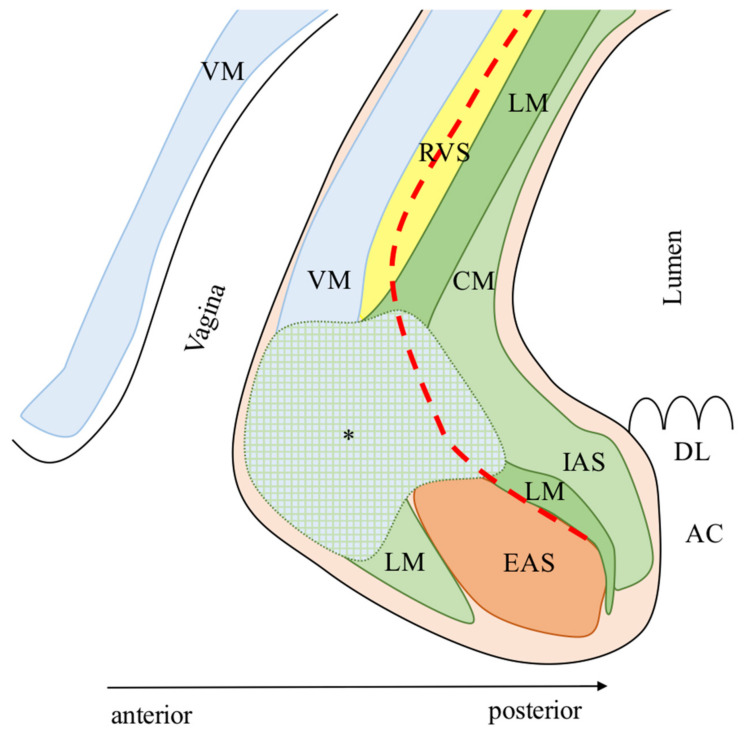
Female’s anterior anatomy. AC: Anal canal; CM: Circular muscle of the anal canal; DL: Dentate line; EAS: External anal sphincter; IAS: Internal anal sphincter; LM: Longitudinal muscle of the anal canal; RVS: Rectovaginal septum; *: Area of intermingling between muscle fibers of the LM, the VM, and the IAS; VM: Vaginal smooth muscle layer; Red line: Dissection plane during intersphincteric resection. The anatomic model was designed according to the descriptions of Muro et al. [33]. Modified and reprinted with permission from ref. [20]. Copyright 2020 Turkish Journal of Colorectal Disease.

The mucous membrane of the distal rectum/anal canal, the IAS, the LM, and the EAS are found from the lumen outward in both genders (Figure 2 and Figure 3). The key structure for understanding the anterior region is the LM with its different spatial distribution between genders. 

In males, LM fibers divide caudally into three bundles on the sagittal plane: (1) posterior to the EAS, covering the anterior surface of the IAS; (2) anterior to the EAS, forming the anterior bundle of the LM (AB), which is sandwiched between the bulbospongiosus muscle (BM) and the EAS and terminates into loose connective tissue; (3) anteriorly forming the rectourethralis muscle (RU) [32,34]. 

In females, LM fibers divide caudally into two bundles: (1) the medial fibers of the LM run downwards and, together with the IAS, converge anteriorly merging into the posterior vaginal smooth muscle layer (VM), the vaginal vestibule, and the perineum covering the anterior surface of the EAS and forming an anterior dense area of muscular intermingling; (2) the lateral fibers of the LM instead run medially between the EAS and IAS [33]. 

The ISP is not clearly evident in the anterior region in both genders but can be found through two anatomical landmarks. In males, the anterior dissection is performed along the Denonvilliers’ fascia (anteriorly or posteriorly according to the anterior tumor’s extension for a safe circumferential resection margin). The dissection then proceeds caudally along the LM fibers and through the posterior portion of the RU muscle. This phase is crucial. Care must be taken not to cause rectal perforation posteriorly and urethral injuries anteriorly. Moreover, the RU must be dissected in its most caudal portion to avoid injuries of the cavernous nerve [35,36]. The RU runs anteriorly, in the upper part to the urethral rhabdosphincter, and in the lower part directly to the membranous urethra, therefore is a crucial dangerous area during dissection [34]. Because of the deep and anterior location of the RU, its visualization and dissection are challenging through open and laparoscopic approaches, according to the authors’ experience. The robotic platform, with a magnified three-dimensional stereoscopic view, optimizes the identification of the RU that appears as small bundles of fibers detaching from the LM and running anteriorly on a plane parallel to the pelvic floor [21]. 

The RU muscle is a key anatomical element of the male deep pelvis. The RU does not only have a tight connection with the LM posteriorly but also spreads laterally sandwiching the LAM: superiorly to the LAM forming the above-mentioned HL; inferiorly to the LAM (attaching to the ischiopubic ramus between the obturator internus and the ischiocavernosus) forming the deep transverse perineal muscle [34,36]. 

In females, the anterior dense area of muscular intermingling, which is the deep caudal endpoint of the rectovaginal septum (RVS), is visible only after a correct dissection of the RVS. This anatomical landmark can be easily visualized through open, laparoscopic, and robotic approaches, according to the authors’ experience, as the terminal anterior dense tissue between the rectum and the vagina. This dense tissue is dissected to complete the ISR, taking care not to perforate the rectum posteriorly and the vagina anteriorly.

The Denonvilliers’ fascia and the RU are the anterior landmarks for the correct anterior dissection in males during an ISR, while the RVS and the anterior dense area of muscular intermingling are for the females. Because the anterior region misses a clear surgical plane, the anterolateral region of the rectum should be previously dissected to optimize the anterior dissection during ISR [21,33].

### 2.3. Lateral Anatomy

The structures of this region are very similar to the posterior region, with three differences [30]. First, the smooth muscle layer covering the LAM and forming the lateral extension of the HL (or endopelvic fascia) is very thin in this portion. Second, the length of the attachment between the LM and the LAM decreases significantly in an anterior to posterior direction, with the anterolateral having the greater extension. Third, the overlap between the LAM and the EAS increases as it moves posteriorly [30]. The surgical landmark for a correct lateral dissection is the plane found between the medial edge of the LAM (that can be easily identified through muscle contraction with electrocautery) and the rectum [21].

## 3. Definition of ISR

ISR is an anus-preserving technique for LRC described by Schiessel et al. [18] in 1994 as a combination of two techniques: the intersphincteric rectal excision for inflammatory bowel disease [37] and the coloanal anastomosis for low rectal resections [38]. ISR is characterized by two distinct phases: abdominal and perineal. ISR allows extension of the caudal dissection plane to allow a safe distal margin for very low-lying rectal cancer without excising the sphincter complex (EAS/LAM) as in the APR. The oncological safety of the ISR derives from the knowledge that lymphatic spread of low rectal cancers occurs especially in the oral direction within the mesorectum with local spread present only in few millimeters [39,40]. 

**Figure 4:**
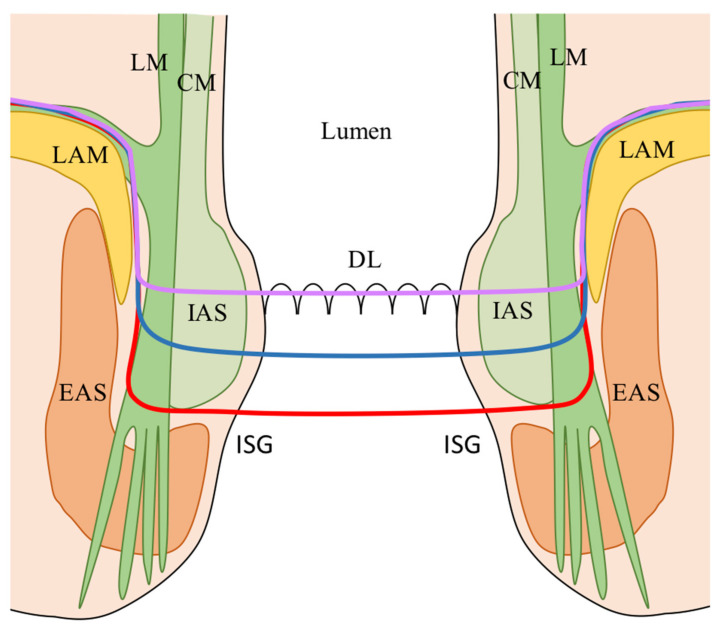
ISR classification according to the extension of dissection [41,42]. Red line: total ISR (complete resection of the IAS at the (ISG); Blue line: subtotal ISR (resection line between the DL and the (ISG); Purple line: partial ISR (resection at the level of the (DL). CM: Circular muscle of the anal canal; DL: Dentate line; EAS: External anal sphincter; IAS: Internal anal sphincter; ISG: Intersphincteric groove; LAM: levator ani muscle; LM: Longitudinal muscle of the anal canal.

ISR was originally classified as subtotal and total according to the partial or complete resection of the IAS [18]. However, the Japanese experience on ISR has modified the original classification into three types (Figure 4) [41,42]: (1) Total ISR (complete removal of the IAS at the intersphincteric groove); (2) Subtotal ISR (the resection line lays between the dentate line (DL) and the (ISG); (3) Partial ISR (the resection is at the level of the (DL). The choice of the dissection line depends on the lower border of the tumor in order to obtain an adequate distal clearance (distal resection margin (DRM) ≥ 1 cm). 

The intersphincteric dissection for ISR is usually started in the transabdominal phase and completed during the perineal phase [19,43,44,45,46,47,48,49,50,51,52,53,54,55,56,57]. However, some authors perform the intersphincteric dissection in toto only during the perineal phase [58,59,60,61,62,63,64,65,66]. Park et al. and Kim et al. have reported the use of the robotic platform to perform total/near-total single-stage transabdominal ISR with no need for a perineal intersphincteric dissection [67,68]. 

## 4. Indication of ISR

ISR is a surgical technique for treating patients with LRC, generally defined as tumors with the caudal edge within 4–5 cm from the AV or 2 cm above the DL. Surgical indications for ISR have changed since the standardization of the technique [18] (Table 1). However, a precise preoperative staging with the combination of rectal MRI, thoracic-abdominopelvic computed tomography (CT) scan, anal EUS, rigid proctoscopy, and digital rectal examination (DRE) remains crucial for a correct surgical indication [69]. Restaging should be done after nCRT, and surgical indications must be always re-checked and re-discussed with the patient. The final decision to perform an ISR or convert to APR is done by the surgeon in the operating theatre, before starting surgery, while performing DRE under anesthesia to access tumor mobility and relationship to the anal sphincters [49,60].

The reviews of Martin et al. [69], Akagi et al. [41], and Shirouzu et al. [70] have critically discussed the indication criteria for ISR. They all agreed that ISR should be indicated for well/moderately differentiated T1–3 tumors located within 5 cm from the AV, independently to IAS invasion. Poorly differentiated, T4, fixed tumors (at DRE), with EAS/LAM infiltration and/or untreatable distant metastases should be contraindicated to ISR. Moreover, ISR should not be indicated to patients with poor anal function, severe preoperative pathologies (cardiac failure, liver cirrhosis, renal dysfunction, and respiratory dysfunction), or psychiatric disease. These indications were confirmed by a national-based questionnaire from the Japanese Society for Cancer of the Colon and Rectum (JSCCR) [71]. 

Rullier et al. implemented the indication criteria for LRC, through an MRI-based classification [72]. They classified LRC into four types to assist decision-making between anus-preserving surgery and APR. Type I are LRC defined as supra-anal tumors (lesions >1 cm from the anorectal ring). Type II are defined as juxta-anal tumors (lesions ≤ 1 cm from the anorectal ring). Type III are defined as intra-anal tumors (lesions with IAS invasion). Type IV are defined as transanal tumors (lesions with EAS or LAM invasion). They indicated ISR for type II and III. Furthermore, some authors have proposed to combine ISR with partial resection of the EAS for type IV tumors [42,73,74]. Rullier’s classification is very intuitive and of easy use, however, it evaluates the tumor position, from MRI images, in relation to the LAM and EAS in a frontal view without considering the circumferential position of the tumor on the anal clock. Through a retrospective analysis of surgical specimens, Kang et al. analyzed the circumferential tumor location, reporting that the anterior aspect most frequently involves the circumferential resection margin (CRM) and exhibits a deeper tumor invasion [75]. Further studies are needed to define if the circumferential tumor location may in the future play a role in the treatment strategy, such as stronger indication for preoperative radiotherapy or choice of surgical approach.

The indication criteria for ISR have been recently discussed by two studies. Park et al. [56] have examined the role of the tumor’s response to nCRT on restaging pelvic MRI as indication criteria for ISR. They reported the ymrT stage and ymrCRM status as key factors for deciding between ISR and APR. Moreover, ISR indication was extended also to patients with cT4 LRS that downstaged after CRT (i.e., ymrT0–3). Poor responders (i.e., ymrT3) with suspicious tumor invasion of the CRM should instead undergo APR.

Piozzi et al. reported a study on 161 ISR where indication criteria were extended. In this study patients with post nCRT clearance of EAS/LAM infiltration were indicated to ISR independently to T stage if curative resection was considered technically feasible at the pre-operative MRI staging [57]. 

Clinical indications to ISR have changed in the last three decades however an international consensus should be taken to critically define them in order to perform standardized ISR with comparable results throughout the colorectal community.

## 5. Distal Resection Margin and ISR

The distance between the tumor and the anal sphincters has been for a long time the main indication for anus-preserving resection, following the potential risk of leaving microscopic residual disease distally to the tumor responsible for LR [76]. The traditional distal margin of 5 cm for rectal resections was reappraised in the 1980s for the shorter 2 cm allowing a potential increase in anus-preserving resections [77,78]. However, considering the short length of the anal canal (3–4 cm) [45], only APR has been the standard surgical treatment for very LRC for both technical and oncological reasons. ISR allows, through the partial or total excision of the IAS, to extend and maximize the distal resection margin (DRM). Rullier et al. advocated the end of the 2 cm distal rule through their series of 98 patients (85% of which ≥pT3, 20% with IAS involvement, and 88% submitted to nCRT) by reporting 81% of 5 years OS with 2% of LR (median FU: 40 months) and a 2 cm DRM negative in 98% of cases [45]. 

Considering that LAM, EAS, ischiorectal tissue, and perianal skin are rarely involved by rectal tumors, even when poorly differentiated, and that the intraluminal and mesorectal spread rarely exceeds 1–2 cm, the DRM was re-discussed [79]. The DRM was reduced to 1 cm (with or without preoperative nCRT) [80,81,82,83] with some reports even advocating for a shorter DRM [40,84,85]. Moore et al. also reported no difference in outcome for patients with DRM greater than or less than 1 cm in the setting of nCRT [81].

A study from the Norwegian Colorectal Cancer Registry on 3571 patients undergoing rectal resection reported a negative impact on tumor recurrence when the DRM was <10 mm [83], therefore, a DRM ≥ 1 cm (on the fresh specimen) should be considered safe for ISR. 

Krand et al. reported that when the perioperative frozen section analysis reported a DRM < 1 cm, they did not perform a re-excision unless the DRM was positive/suspicious, in order to preserve at least 50% of the IAS mass [59]. On a total of 47 patients, one had a DRM of 3 mm and seven of 8–9 mm (all yT2), none of them developed an LR. This result confirmed an older study of Karanjia et al. [86], demonstrating that shorter DRM, in selected patients (post nCRT and low T stage) is oncologically safe.

It is important not to underestimate the specimen shrinkage after manipulation for pathologist evaluation. Formaldehyde fixation reduces the DRM up to 57% after 12–18 h [87]. Therefore, it is important to always consider the DRM on the fresh specimen for surgical reports.

Thus, during ISR the surgeon’s goal is to provide a macroscopic DRM of 1 cm. However, a DRM below 1 cm is considered safe at the final pathological examination if the margin is free (Table 2) [40].

## 6. Circumferential Resection Margin and ISR

CRM is key for oncologically safe rectal surgery [91,92]. The Dutch study, including 181 patients with 17% of positive CRM, clearly demonstrated a higher 2 years LR rate in positive CRM compared to negative CRM patients (13% vs. 4%) [93]. Moreover, CRM was found to be associated not only with LR but also with distant metastases [94,95,96,97,98].

The CRM cut-off for ISR is the same as for standard proctectomy (CRM ≤ 1 mm) [99,100]. However, the mesorectum in the low rectum is very thin or nearly absent. After invading the muscularis propria the tumor can extend into the ISP and infiltrate the striated muscles of the pelvis (EAS and LAM). Because ISR is performed through the ISP, medially to the striated muscles, an invasion of EAS/LAM is considered a technical contraindication [45], with only a few authors advocating for partial EAS excision in specific cases [42,73,74]. The evaluation of ISP tumoral clearance is of utmost importance and mandatory for ISR indication. ISP clearance is assessed through preoperative rectal MRI and DRE, both at the time of clinical staging and before starting surgery (after anesthesia induction) in order to access tumor mobility and tumor relationship to the anal sphincters [49,60].

Crucial for CRM assessment is not only the surgical procedure but also the slicing method performed by the pathologist. The transverse slicing method for CRM assessment is the most common in western countries [91,101], however, the Japanese Classification of Colorectal Carcinoma recommends the longitudinal slicing method [55]. Matsunaga et al. performed a detailed report of this method on 197 patients submitted to ISR [55]. They recognized two different types of CRM-positive patients. The DEEP group (70%) where CRM was positive at the longest distance between the tumor surface and the outermost part, and the ENTRY group (30%) where CRM was positive between the initial cutting point (at the anal side) and the deepest tumor invasion. Interestingly, the ENTRY group was associated to lower (*p* = 0.021) and poorly differentiated (*p* = 0.013) tumors. This provides important feedback to surgeons to properly perform distal dissection and to take particular attention to poorly differentiated tumors. 

ISR allows for oncologically safe sphincter preservation with good CRM rates (Table 2).

## 7. Neoadjuvant CRT and ISR

LR control is the main concern for rectal surgeons, especially for very low-lying tumors. The oncological safety of ISR has been long criticized by colorectal surgeons in favor of the traditional APR, especially regarding LR control. LR rate after ISR has been reported to range between 0–22.7% [70]. This wide range depends on the great variability in the surgical series between the 1990 s and 2021 (Table 2). One of the most significant differences between the studies is the cT stage and the rate of nCRT (0–100%) [70]. The wide difference in nCRT adoption follows the controversies regarding the ability to increase or not the sphincter preservation rate.

nCRT allows downstaging and downsizing of the lesion (up to 56% of patients with tumor regression grading 3–4) [102], reduction in tumor deposits, budding, and micrometastases [103]. Moreover, up to 20% of complete responses (depending on the waiting period) have been reported [10,11,12].

nCRT is a standard treatment to reduce positive CRM and LR rate in locally advanced rectal tumors according to the NCCN guidelines [15,104,105].

Interestingly, in Japan, nCRT is not indicated for resectable cT1–3 tumors with suspicious lymph nodes if located within the TME boundaries [70]. The safety of this strategy was showed by Akagi et al., who reported a low rate of LR (4.8%) with a 5 years survival of 76–97% (ISR without nCRT) [53]. There is also a strong concern on the consequences of nCRT for patients undergoing ISR such as higher surgical complications [15], negative impact on anal [106,107], urinary [108], and sexual function [109] with no clear survival benefit [104].

In the authors’ center, nCRT is performed when the staging rectal MRI reports a threatened or suspicious CRM and/or in the presence of LN >5 mm in short-axis diameter on the lateral pelvis outside the TME plane. This allowed an LR of 5.6% after 2 years, in range with published literature [110,111,112].

Some authors advise against indicating ISR to patients with infiltration of EAS/LAM, with tumor shrinkage after nCRT, because of the possibility of unexpected residual malignancies [62]. However, recent studies advocated the indication to ISR if the EAS/LAM infiltration was not evident after nCRT at the restaging MRI [56,57]. Moreover, Park et al. reported that the oncological outcomes in their series of ISR after nCRT were less related to the clinical stage showing stratification according to tumor response (for ypT3 3-y DFS 47.4% and 3-y LR 15.1%) [56]. The authors concluded that APR might be more beneficial for patients who do not show downstaging after nCRT. 

Piozzi et al. reported a study of 161 patients submitted to ISR including locally advanced tumors (ypT4; *n* = 6) not responsive to nCRT [57]. Between patients with LRs, two were anterior ypT4 that underwent radical ISR resection with negative margins (R0) and negative intraoperative frozen biopsies (outside the boundaries of the ISP). Therefore, ypT4 patients should be evaluated with great attention and APR should be considered especially for cases with anterior involvement, where radical clearance can be more challenging.

## 8. Surgical Approach: Open vs. Laparoscopic vs. Robotic

Compared to open surgery, the laparoscopic approach provides improved early postoperative outcomes including reduction in intraoperative blood loss, postoperative pain, ileus rate, and better cosmesis leading to earlier recovery and hospital discharge [113,114,115,116,117,118]. In addition, the laparoscopic approach to rectal cancer was reported to be feasible and oncologically safe with no differences in overall (OS) or disease-free survival (DFS) when performed by highly experienced colorectal surgeons [113,114,115,116,117,118]. However, the laparoscopic approach to rectal cancer is technically challenging because of unarticulated straight rigid instruments, an assistant-dependent, unstable, two-dimensional unmagnified view, and poor ergonomics in the narrow deep pelvic cavity [113,119]. These technical flaws may be the cause of the steeper learning curve of laparoscopic compared to open surgery and be responsible for traction injuries, rectal cross stapling, difficult retraction, and crowding of instruments causing suboptimal clinical results for inexperienced surgeons. 

The da Vinci^®^ Surgical System (Intuitive Surgical Inc., Sunnyvale, CA, USA) was engineered to overcome the technical limitations of laparoscopy by providing better ergonomics, eliminating physiologic tremors, adding an extra working arm, improving dexterity (articulated instruments with seven degrees of freedom), and introducing a surgeon’s controlled magnified three-dimensional stereoscopic stable camera [120]. 

The technical improvements from the robotic platform were indirectly demonstrated by Baek et al., in a report where patients with rectal cancer were stratified into three groups of pelvic difficulty according to preoperative MRI pelvimetry [121]. The authors reported no differences between the groups in terms of surgical outcomes, leading to postulate that robotics has compensated for the level of surgical difficulty. This study strengthens the role of the robotic approach for difficult cases (i.e., post-neoadjuvant treatment, males, LRC) and technically demanding procedures as ISR that require meticulous, efficient traction and excellent vision for accurately identify the anatomical landmarks for an oncologically safe dissection [21,122,123,124,125].

The robotic approach allows experienced hands to even perform a completely transabdominal ISR, by extending the abdominal phase in the ISP, opening into the anal canal lumen, and reducing the perineal time [126]. This was reported to be feasible in up to 86.9% of performed ISR [127]. The robotic approach enables intersphincteric dissection up to the level of the subcutaneous EAS thanks to its enhanced dexterity and magnified field [128]. The dissection into the ISP cannot be safely performed as well, via a transabdominal route, with the open or laparoscopic approach [64,129,130]. Therefore, for the open and laparoscopic approach, a perineal phase is inevitable for completing the ISR and this could lead to possible technical injuries when joining the two different dissection planes (transabdominal and perineal) leading to positive CRM, however, it is not yet demonstrated. 

Few studies have compared open (OISR) to laparoscopic (LISR) and LISR to robotic ISR (RISR) for LRC. A meta-analysis comparing OISR to LISR on eight studies reported the latter to have comparable operative time, less blood loss, shorter length of postoperative hospital stay, quicker time to first flatus, less incidence of morbidity, and pneumonia with comparable pathological and survival outcomes [131]. This report confirms similar results of a previous meta-analysis on five studies performed by Chen et al. [132].

Shin et al., performed a retrospective comparative study between OISR and minimally invasive ISR (LISR/RISR) on a large series of 313 LRC submitted to ISR demonstrating no differences in 5 years OS and DFS also after applying a propensity score matching analysis [133]. 

A meta-analysis on RISR vs. LISR for LRC from Lee et al. [134] analyzed five studies [122,135,136,137,138]. They reported that RISR was associated with a lower conversion rate, lower estimated blood loss, and longer operation time compared to LISR. Moreover, fewer harvested LN were noted in RISR with no statistical significance. Interestingly, other perioperative outcomes, functional outcomes, 3 years survival, and LR were statistically similar between the two groups. However, this study had several limitations, such as including only retrospective cohort studies and no comparative randomized trials and including only studies from two East Asian countries (Korea and Taiwan). However, this geographical bias is consequent to the rapid development of robotic-assisted techniques in East Asia, especially in Korea, compared to the West.

One of the studies included in the meta-analysis was a multicenter study involving seven institutions from the Korean Laparoscopic Colorectal Surgery Study Group [135]. They analyzed the long-term follow-up outcomes with a relatively large patient population to verify the long-term safety of RISR for LRC compared with LISR. They reported no statistical difference between RISR and LISR for cT3–4 tumors on 3 years LR (*p* = 0.930) and 3 years DFS (*p* = 0.887) [135]. However, Korean colorectal surgeons are highly trained and skilled in the laparoscopic approach with often very large minimally invasive series; this bias could have reduced the possible difference between LISR and RISR in perioperative and oncological outcomes.

Kim et al. have published a long-term retrospective study on RISR patients reporting 5 years cumulative rates of LR, OS, and DFS of 2.5%, 86.7%, and 80.7%, respectively [127]. 

Yoo et al., have reported no significant differences between LISR and RISR in the 3 years OS (88.5 vs. 95.2%; *p* = 0.174), 3 years recurrence-free survival (RFS, 75.0 vs. 76.7%; *p* = 0.946), and 3 years local RFS (91.7 vs. 87.2%; *p* = 0.466) [138].

Recently, Kim et al. published a pivotal study showing how the robotic platform facilitates an efficient sphincter-saving resection in patients with LRC by comparing RISR to OISR [128]. The robotic approach was reported to be the most significant parameter for ISR achievement (OR: 3.467; 95% CI: 2.095–5.738; *p* < 0.001). Moreover, the robotic approach was associated with higher rates of subtotal/total ISR (advanced ISR) compared to the open approach (47.8% vs. 20.2%, *p* < 0.001) with a significantly lower anastomotic level (*p* < 0.001). There was no difference in pathological stage, histological differentiation, DRM length, and CRM positivity between RISR and OISR. Besides longer operative time (*p* < 0.001), perioperative outcomes were superior in RISR: lower blood loss (*p* = 0.002), lower postoperative pain score (*p* = 0.02), lower extra-analgesic use (*p* = 0.001), lower general surgery complications (16 vs. 7.7%, *p* = 0.001), lower voiding difficulty (*p* = 0.02), lower sexual dysfunction moderate-to-severe grades in males ≤65 years (34.8 vs. 17.2%, *p* = 0.03), lower permanent stoma retention (*p* = 0.04), faster bowel passage (*p* < 0.001), lower gas incontinence score (*p* < 0.01), lower fecal incontinence score, and lifestyle alteration score (*p* < 0.05). However, the functional outcomes difference might also be related to higher nCRT rates in the OISR group (80.6 vs. 48.4%). No statistical difference was reported in the oncological outcomes between RISR and OISR: 3 years cumulative LR rate (3.6 vs. 3.8%, *p* = 0.96), 3 years cumulative systemic recurrence rate (17 vs. 14.4%, *p* = 0.58), 3 years OS (91.1 vs. 90.4%, *p* = 0.89), and 3-y DFS (79.5 vs. 79.8%, *p* = 0.67). The authors reported also a nil conversion rate in the RISR group, which can be associated with the high experience of the operator (30 years experience with 4000 rectal cancer operations). Conversion rate is lower compared to published LISR rates (0–21.8%) [122,130,139,140] and similar to other RISR studies [57]. In addition, the operators’ physical discomfort was significantly lower in RISR (*p* < 0.001) on a visual analog scale; this should be carefully considered as an important factor considering the high technical and psychophysical demand of ISR for the surgeon.

RISR allows to significantly replace APR, in experience hands, to achieve a successful anus-sparing resection for LRC. Long-term well-designed multicentric randomized controlled trials, comparing LISR and RISR, are required to confirm the advantages of the robotic approach for anus-sparing LRC resections. 

Recently Kim et al. reported the first case of ISR performed with the da Vinci^®^ Single-Port (SP) System (Intuitive Surgical, Sunnyvale, CA, USA) (SP-ISR) on a 73-year-old male with a ypIIIC rectal cancer at 5 cm from the AV [141]. Moreover, Cheong et al., have performed an SP-ISR on a 57-year-old female patient with a ypT2N0M0 (0/20; CRM = 7 mm) rectal cancer at 3 cm from the AV [142]. This new robotic platform is characterized by a single-arm that can rotate 360° allowing multi-quadrant anatomical access without the need for redocking. The instruments are all located within the single-arm and have an additional elbow joint proximal to the wrist joint for triangulation and independent planar movement of the instrument tips to avoid collisions. The single-incision for the SP system is located at the right lower quadrant. This position is optimal for multi-quadrant access for ISR, and it is used for specimen extraction and stoma site. However, the SP system does not have a vessel sealer, suction, or stapler so an additional trocar is still required. Future studies with extended series are needed to outline the outcomes of SP-ISR.

## 9. Risk Factors for Oncological Outcomes after ISR

Few studies, all from Korea, have analyzed the risk factors for LR, OS, and DFS after ISR.

Lee et al., retrospectively reviewed 163 patients with LRC, without distant metastasis, who underwent OISR/LISR following nCRT. They compared the outcomes of patients undergoing OISR or LISR. They reported 3 years DFS (stage 0, 96.2%; I, 84.8%; II, 72.9%; III, 38.0%) and 3 years LR-free survival (stage 0, 100.0%; I, 92.4%; II, 91.1%; III, 70.9%) after a median follow-up of 53 months (range 0–82 months). The authors reported ypT (*p* = 0.006; HR: 2.947, 95% CI: 1.370–6.339) and ypN (*p* < 0.001; HR: 3.282, 95% CI: 1.714–6.283) stages as prognostic factors for DFS. Tumor height (2 cm cut off; *p* = 0.001; HR: 7.385, 95% CI: 2.281–23.917), tumor size (3.5 cm cut off; *p* = 0.002; HR: 5.267, 95% CI: 1.864–14.883) and ypN stage (*p* = 0.014; HR: 3.487, 95% CI: 1.294–9.394) were reported as prognostic factors for local recurrence-free survival [143]. 

Park et al. retrospectively reviewed 147 patients with LRC, without distant metastasis, undergoing LISR/RISR after nCRT [56]. The authors have identified the CRM involvement (*p* = 0.027; HR: 2.361, 95% CI: 1.102–5.060), the ypT stage (*p* < 0.001; HR: 4.681, 95% CI: 2.295–9.546), the ypN stage (*p* = 0.018; HR: 2.258, 95% CI: 1.153–4.423), and the ymrT stage (*p* = 0.043; HR: 2.01, 95% CI: 1.021–3.965) as predictors of cancer relapse after a median follow-up of 34 months (range 8–94 months) [56]. 

Piozzi et al., retrospectively reviewed 161 patients with LRC, including distant metastasis and advanced T4 patients, undergoing LISR/RISR [57]. The authors reported a 5-y OS of 80% and a 5-y LRFS of 87%, after a median follow-up of 55 months. Preoperative CEA (*p* < 0.001; HR: 4.453, 95% CI: 2.015–9.838) was a prognostic factor for OS. Despite not statistically significant, tumor size (*p* = 0.056; HR: 2.546, 95% CI: 0.976–6.637) and locally advanced T-stage (*p* = 0.062; HR: 3.296, 95% CI: 0.941–11.548), especially with anterior involvement, should be carefully evaluated for optimizing the surgical strategy (LISR/RISR vs. APR). This study confirmed the oncological safety of ISR for advanced low-lying rectal tumors by also including patients with locally advanced tumors not responding to nCRT. This was not considered a contraindication to ISR if curative resection was technically feasible at the preoperative MRI staging (no evidence of involvement of the EAS/LAM after nCRT). Interestingly, Park et al. have also discussed the role of the tumor’s response to nCRT on restaging pelvic MRI as indication criteria for ISR [56]. The authors included cT4 that downstaged after nCRT, and advised not to consider ISR for poor responders with suspicious CRM positivity. The authors also reported post-radiation MRI T stage as a good predictor for both LR and DFS [56]. 

Kim et al. have extended the TME concept of resection completeness for surgical quality to ISR through the definition of total intersphincteric longitudinal muscle excision (TILME) [68]. TILME indicates the en-bloc excision of anorectal tissue adjacent to the EAS including the LM and intervening fibro-fatty tissue. TILME quality was based on the completeness of LM excision and depends on two factors: LM defect > 5 mm and CRM ≤ 1 mm (evaluated by longitudinal section of the specimen [55]). The authors reported a rate of complete-TILME in 84.5% of patients, which was lower in the total vs. partial/subtotal ISR group (*p* < 0.05) and correlated to the completeness of TME (*r* = 0.0283, *p* < 0.001). Completeness of TILME resulted to be the only risk factor for 5-y cumulative LR (OR: 23.385, 95% CI: 1.492–366.421; *p* = 0.03). Moreover, 5 years DFS and OS were higher in patients with complete-TILME, however, with no statistical significance. Therefore, ISR observing complete TILME can reduce the LR. The robotic platform with its technical advantages could improve TILME completeness rates, however further studies are needed. Moreover, the concept of TILME completeness has to be carefully considered along with the anatomical features of the LM (which is variable in thickness) and the presence of adjacent fat separating the LM from the EAS in the ISP [23]. 

## 10. ISR vs. APR

ISR was introduced as the ultimate anus-preserving technique for LRC and can be considered as a longitudinal extension of a beyond-TME procedure [144]. ISR is an alternative to APR for very low-lying rectal cancer. Some authors have compared the clinical and oncological results between ISR and APR, however, this should be carefully considered because the indications for these two techniques do not always overlap [145]. The ISP can be generally considered the anatomical boundary between these two techniques with ISR indicated for tumors not infiltrating the EAS/LAM complex. The other main factor is the presence of preoperative anal sphincter incontinence which is an absolute contraindication to ISR and indication of APR. Furthermore, poorly differentiated adenocarcinomas are an indication of APR. Therefore, patients submitted to ISR are usually younger, with a lower pT stage [145]. As mentioned above, ISR is indicated for type II and III rectal cancers according to Rullier’s classification [72], while APR is indicated for type IV. Moreover, there is also a technical aspect that should be considered when comparing ISR to APR. ISR is an anatomically planned technique with specific well-described steps and anatomical landmarks [19,21]. Only recently, APR was standardized into three techniques according to well-defined anatomical structures to improve the oncological results: intersphincteric, extralevator, and ischioanal APR [3,146,147]. Therefore, when ISR is compared to APR, it should be specified which type of APR was performed.

Peng et al. performed a meta-analysis on 12 studies comparing ISR and APR for LRC [145]. The authors reported no significant differences in gender, body mass index, distance from tumor to the anal edge, operative time, and blood loss. Hospital stay (−2.98 days, *p* < 0.00001) and postoperative morbidity (*p* = 0.04) were significantly lower in the ISR group, following the smaller perineal wounds. Despite the ISR group had a lower pT stage (*p* = 0.01), the 5 years OS, 5 years DFS, and 3 years LR rates were similar between the two groups. 

As aforementioned, ISR was often criticized to be not as oncologically safe as APR, especially for LR control. Saito et al., have compared ISR to APR and reported similar results in 5-y local relapse-free survival (83% vs. 80%; *p* = 0.364), 5 years DFS (69% vs. 63%; *p* = 0.714), and LR rate (10.6% vs. 15.7%; *p* = 0.295) [148]. However, there was a significant difference between the ISR and APR in margin recurrence rate (3% vs. 11.4%; *p* = 0.017) and 5 years OS (80% vs. 61.5%; *p* = 0.033) [148]. 

Okamura et al. compared the oncological results of ISR and APR by analyzing the patients from a multicentric study of the Japan Society of Laparoscopic Colorectal Surgery to specifically evaluate the local control rate between these two techniques [149]. The analysis was performed only on advanced tumors (T3–4, any N, M0) located 2–5 cm from the AV that would be eligible for both ISR and APR. After performing a propensity score analysis to adjust confounders, the authors reported no differences in 3 years LR rate between ISR and APR (11 vs. 14%; HR: 0.77, 95% CI: 0.42–1.41; *p* = 0.40). They also reported no differences in CRM involvement rates between ISR and APR (4 vs. 6%; HR: 0.58, 95% CI: 0.23–1.45, *p* = 0.35) confirming the oncological safety of ISR.

Tsukamoto et al. performed a retrospective propensity score-matched study comparing ISR to APR [150]. The propensity score analysis was performed to minimize selection bias between the two groups. After matching, they reported similar 5 years RFS (69.9% vs. 67.9%; *p* = 0.64) and 3 years cumulative LR rate (7.3% vs. 3.9%; *p* = 0.13) between ISR and APR. 

The APR rate is heterogeneous between surgeons and countries with still a high rate of patients with LRC undergoing such extensive procedures (29 to 40%) [151,152,153]. However, Kim et al. recently reported that since the introduction of ISR, the APR rate dropped to 4.9% with a rate of 1.2% in the last fourth quarter of the study [68]. Moreover, according to the authors, there were no differences in the selection of operative indications during the study period with overlap between the APR and ISR. 

Rouanet et al., have reported the oncological long-term (10-year) results from the French multicenter, prospective, randomized trial GRECCAR 1 [154]. This trial, which started in 2001, analyzed the long-term results of patients initially indicated to APR who were submitted to nCRT and anus-sparing resection (mucosectomy and CAA, partial ISR, or complete ISR). The total anus-preservation rate was 84.6% (72% ISR) with good 10 years OS (around 70%), 10 years LR (10.2%), and 10-y distant metastases rate (27.6%). Moreover, 10 years OS and DFS were significantly longer for anus-sparing resections (72.2% and 60.1%, respectively) compared to APR (54.7% and 38.3%, respectively). This trial demonstrated the feasibility of safely changing an initial APR indication to an anus-sparing resection such as ISR according to the tumor response to nCRT.

These results show that ISR is an oncologically safe anus-preserving alternative to APR and that many patients could benefit from it. A prospective randomized trial is necessary to confirm these results, however, it would be ethically unfeasible to randomize patients without considering their decision or not to preserve the anus. Therefore, an extensive multicenter study with standardized indications, technique, approach, and nCRT regimen is needed to evaluate the oncological results of patients undergoing ISR who otherwise would have undergone APR confirming the GRECCAR 1 results.

## 11. Patterns of LR after ISR

Few studies have reported the characteristics of LR and their locations after ISR.

Akasu et al. reported a total of 6 (5.7%) LR in a series of 106 patients with T1–3 LRC not submitted to nCRT [50]. The LR were located: internal iliac/obturator nodes in three patients, circumferential resection margin in two patients, and sacrum in one patient. The authors reported pT3 and positive microscopic resection margin as risk factors for LR.

Beppu et al. reported a total of 6 (5.8%) LR in a series of 104 patients [155]. They reported four lateral and two central LR. Interestingly, on seven patients with positive CRM, only two experienced LR.

Shin et al. reported a total of 14 (4.5%) LR in a series of 313 LRC (eleven LR at the pelvic wall and three at the anastomotic site) [133]. 

Park et al. reported a total of 17 (11.6%) LR in a series of 147 patients [56]. The authors documented the location of the LR (ten lateral, four axial (anastomotic site), two presacral, and one at a port site) but did not report the patients’ characteristics.

Piozzi et al. documented a total of 18 (11.1%) LR in a series of 161 patients including locally advanced tumors (ypT4) not responsive to nCRT [57]. They reported nine axial, five lateral (one with a suspicious LR on the anastomotic site), two presacral, and two anterior according to the Dutch TME trial classification [156]. Interestingly, only one patient with LR had a positive CRM at the time of the primary surgery. Two patients with a ycT4 tumor with preoperative suspect of anterior invasion underwent radical ISR resection, but despite negative margins (R0) and negative intraoperative frozen biopsies (outside the boundaries of the ISP), they developed an LR. Therefore, post-radiation cT4 patients should be evaluated with great attention and APR should be considered especially for cases with anterior involvement, where radical clearance can be more challenging.

Sato et al. performed an impressive study evaluating the anorectal lymphovascular networks and tissue drainage to better understand the tumoral spread outside the rectal wall [157]. Through the use of submucosal India ink injection into fresh cadavers and resected specimens, and intra-operative indocyanine green fluorescence imaging they draw the anorectal-pelvic lymphovascular pathways. They reported a tight relationship between the anorectum and the pelvis through evidence of dye disseminated in the stromal tissue around blood vessels in the LAM fascia, HL, and EAS. These routes could be possibly responsible for LR even when CRM involvement is negative, reinforcing the role of lymphovascular invasion as an important risk factor. Despite not analyzing the anterior portion because of the specimen characteristics, this study outlines the need to better standardize the ISR technique promoting posterior and lateral clearance of the HL (and endopelvic fascia) above the LAM together with the posterior dissection of the RU (in males) in a smooth muscle-including ISR. 

Future multicenter studies focusing on the patterns of LR are required to better define the indications between ISR and APR. 

## 12. Considerations on Functional Outcomes after ISR

ISR is characterized by preservation of the anus and restoration of bowel continuity. However, ISR is performed through an extensive pelvic dissection and mobilization from the pelvic floor (EAS/LAM) with partial/near-total/total excision of the IAS and the creation of a hand-sewn CAA. Therefore, ISR is characterized by a certain degree of sphincter function impairment [48,158]. Denost et al. performed a long-term retrospective study evaluating the functional outcomes of 171 patients (56%) with a median follow-up of 4.6 years (range 1.0–15.6) [159]. The authors reported a median Low Anterior Resection Syndrome (LARS) score of 30 months (range 0–42) with 44% no LARS, 14% minor LARS, and 42% major LARS. The median continence score was nine (range 0–20) with 32% good continence, 25% moderate incontinence, and 44% major incontinence. Overall, 12% required a definitive stoma. The authors reported an improvement in the functional results following the adoption of an institutional bowel rehabilitation program.

Yamada et al. evaluated the functional results from a JSCCR questionnaire for the standardization of ISR in Japan [71]. The postoperative function of 990 patients at 12–24 months after ISR was evaluated and compared to the systematic review of Martin et al., [69]. Mean daily bowel frequency was 5.0 ± 4.0 vs. 2.7 ± 0.6 of the review. Anal incontinence (Kirwan’s grade III, IV, V) was 37.7% vs. 29.1% of the review. The authors also reported their institutional functional results at 12 months after ISR (*n* = 168). No difference in bowel frequency was evident according to the ISR type. Multivariate analysis of the risk factors for postoperative fecal incontinence indicated age (*p* = 0.004), sex (*p* = 0.047), nCRT (*p* = 0.030), and operative approach (open/laparoscopic vs. robotic; *p* = 0.012) as risk factors according to the JSCCR questionnaire, while the type of ISR (*p* = 0.025), and neorectum reconstruction (*p* = 0.022) were risk factors according to the institutional analysis. 

Kim et al. [127] reported no differences in mean fecal incontinence score (FIS) between partial and subtotal ISR but between total and partial/subtotal ISR at 12–24 months follow-up, confirming a significant functional worsening after complete excision of the IAS. The authors also showed a significant decrease in mean manometry values after ISR, however, continence was recovered by most patients after 12–24 months of follow-up. Maximal tolerance volume and urge to defecate volume were not different between subtotal/total ISR confirming that preserving the DL as much as oncologically possible is clinically relevant. Partial excision of the EAS did not influence the manometry values except for mean resting pressure, which is dependent mainly on the IAS.

Despite sphincter function may gradually improve in the majority of patients after ISR, some may experience worse functional outcomes after stoma reversal. Celerier et al. performed a retrospective study evaluating the risk of definitive stoma formation at a median of 69 months (range 0–232) follow-up [160]. The authors reported 18% (33/180) of definitive stoma in patients submitted to ISR. Anal incontinence was the cause of definitive stoma in 11 cases (33% of all definitive stoma). Interestingly, ISR was not associated with a higher incidence of definitive stoma formation compared to conventional CAA or low colorectal anastomosis (LAR). 

Since the oncological safety of ISR is well documented, postoperative bowel function and overall quality of life should become the main subjects of interest in future studies. Postoperative quality of life should be thoroughly discussed with the patient during surgical indication with care to explain the symptoms and high risks of developing LARS.

Future standardized multicenter studies should be performed on a large number of patients to better understand the functional outcomes with care on separating hand-sewn from mechanical CAA techniques and on evaluating each ISR type (partial, subtotal, or total).

## 13. Learning Curve and Surgical Education on ISR

Learning curve analysis for ISR was performed by Kuo et al., both for laparoscopic [161] and robotic ISR [136]. For LISR, the authors adopted the operating time as a technical indicator for the definition of the learning curve. The authors arbitrarily divided the learning curve into two stages: initial 18 months (*n* = 17) and the following six months (*n* = 11). The authors reported a statistical difference between the two stages in mean operating time (402 vs. 331 min, *p* = 0.034), protective diverting stoma formation (76.5 vs. 0%, *p* < 0.001), and lymph node harvesting (11.1 vs. 18.3, *p* = 0.004). The first stage was characterized by three cases of neorectum necrosis while the second stage had none, however, no statistical difference was reported (*p* = 0.258). Moreover, no statistical difference in clinical characteristics (age, sex, and tumor height), estimated blood loss, postoperative hospital stay, time to first flatus, resumption to oral diet, DRM, CRM, surgical radicality (R0, 1) was reported between the two stages. On multifactorial analysis, surgical expertise (as <18 or ≥18 LISR) was associated with operating time (OR: 2.918, 95% CI: 1.078–7.902, *p* = 0.035) and protective stoma creation (OR: 3.999, 95% CI: 1.153–13.86, *p* = 0.029), but not neorectum necrosis. Age, sex, body mass index, and cT were not risk factors for operating time, protective stoma creation, and neorectum necrosis. Kuo et al. reported that at the time of LISR introduction around 85% of colorectal surgery was performed laparoscopically in their center with around 250 patients treated per year and that all LISR were performed cooperatively by the same two surgeons [161]. Therefore, this learning curve is representative of a surgical team with high expertise in colorectal laparoscopic surgery and cannot be considered for low volumes centers with low laparoscopic expertise.

Kuo et al. [136] constructed the learning curve for RISR using a seventh-order moving average method. The first plateau was observed after 19 patients, which was considered as a turning point between the two stages of the learning curve analysis. The mean operating time was different between the two stages (519 vs. 448 min, *p* = 0.02). No statistical difference was reported in clinical characteristics, estimated blood loss, length of postoperative hospital stay, resumption of oral diet, regaining of bowel peristalsis, DRM, surgical radicality, CRM, mean number of lymph node harvesting, and postoperative complications. On a multifactorial analysis, protective diverting stoma creation and neorectum necrosis were not associated with patients’ age, sex, body mass index, cT stage, or surgeons’ experience. The authors failed to report their previous experience in robotic colorectal surgery before starting to perform RISR.

Despite the limitations of the two studies from Kuo et al. [136,161], it is clear that a certain advanced experience in both laparoscopic and robotic colorectal surgery is required to perform oncologically safe LISR and RISR. Therefore, surgical training in these approaches requires a high surgical volume and the definition of specific professionals in performing such procedures according to the volume. Despite several reports on ISR, there is a lack of educational material for training on such advanced techniques. The reports usually describe surgical series with very little description of the surgical technique. Therefore, to perform an ISR the surgeon needs a proper proctorship program from a trained surgeon which is not always feasible. Following this need, we have previously published a video article aiming specifically to show step-by-step all the anatomical landmarks to perform an oncologically safe ISR [21]. The descriptions in this video article were possible after a thorough review of studies from anatomists, radiologists, and pathologists which are little known to the common surgeon’s circle. It is important that the surgeon rethinks the anatomy of the deep pelvis through the eyes of a pathologist, and this is possible with the enhanced view allowed from the robotic camera. This allows microscopic anatomy to be seen, by experienced surgeons, as macroscopic anatomy. Further educational videos and anatomical reviews are necessary for providing surgeons a precise knowledge of the deep pelvis allowing them to master the ISR technique and reduce the impact of unnecessary APRs.

## 14. Conclusions

ISR is an oncologically safe technique and a valid alternative to APR when indications are carefully selected. Thorough knowledge of the deep pelvic anatomy is of utmost importance to perform high-quality ISR. The robotic approach allows a clear anatomical identification of the dissection plane to optimize the intersphincteric dissection with reported high-quality long-term oncological results.

## Figures and Tables

**Table 1:** Indications for ISR according to the literature.

Authors, Year	Indications	Contraindications
Schiessel, 1994–2012 [18,19,39]	-T1–T3 LRC -Tumor diameter >1 cm -Big villous adenomas -Mucosectomy/RT residual tumors -Low carcinoids/hemangiomas	-Undifferentiated tumors -EAS infiltration -T4 stage -Preoperative insufficient sphincter function -Distant metastases
Vorobiev, 2004 [58]	T2–3 (EUS) Well/moderately diff. adenoca. Fecal continence	-EAS/LAM infiltration -N+ (EUS) -M+
Rullier, 2005 [45]	-≤4.5 cm AV -Distant metastases	-EAS/LAM infiltration -Fixed tumors (except partial vaginal fixity) -Fecal incontinence > 6 months before diagnosis
Hohenberger, 2005 [46]	-≥0.5 cm from DL (rectoscopy) -T1–2 (EUS) -T3 (above puborectal sling) -G1–2 -Patients with possibly distinct invasion of the pelvic floor musculature underwent prior nCRT	-EAS infiltration -Fecal incontinence
Chin, 2006 [47]	-T2 -T3–4 (after nCRT) -≤5 cm (maximal diameter) -1–3 cm from DL	-Distant metastases
Chamlou, 2007 [48]	-T1–3 -T4 if invasion is distant from the tumor’s lowest part/sphincter, and is resectable -Resectable distant metastases -uT1 with adverse pathologic features after transanal local excision	-EAS/LAM infiltration -Fecal incontinence
Krand, 2009 [59]	-(Study on ISR with partial IAS) -Distal excision at the DL or 1–2 mm distal to it -T2–3 -Well/moderately diff. adenoca.	-Total IAS for achieving acceptable DRM -Fecal incontinence -EAS/LAM infiltration -Poorly diff. adenoca. -Distant metastases (except resectable liver metastases)
Han, 2009 [60]	-T1–2 (IAS) -T1-T2 after nCRT -Tumor diameter > 1 cm but <5 cm -Well/moderately diff. adenoca. -Sufficient anal function (DRE, manometry)	-Infiltration of pelvic floor -Tumor diameter > 5 cm -Poorly diff. adenoca. -Insufficient anal function (DRE, manometry) -Distant metastases -Intestinal obstruction
Kuo, 2011 [62]	-T1–3	-Infiltration EAS/LAR (even if submitted to nCRT with radiological clearance)
Martin, 2012 [69] (Review)	-≤1 cm from anorectal ring	-T4 tumors -EAS/LAM infiltration -Fixed tumors at DRE -Poorly diff. adenoca. -Fecal incontinence -Distant metastases
Tokoro, 2013 [52]	-T1–3 -Resectable metastases	-T4 tumors -Poorly diff. adenoca. -Infiltrating gross appearance -Fecal incontinence
Akagi, 2013 [53]	-T1–3 (mobile tumors) -≤4 cm from AV -Well/moderately diff. adenoca. -ECOG PS 0–2 -Good anal function	-T4 tumor -Fixed tumors -Untreatable distant metastases -Poorly diff. adenoca. -Psychiatric disease -Poor anal function (no discernable tone at DRE or the maximum squeeze pressure < 50 mmHg before operation) -Liver cirrhosis, renal dysfunction, cardiac failure, and respiratory dysfunction
Akagi, 2013 [41] (Review)	-T1–3 tumors -30–35 mm from AV -Independently to IAS invasion	-As for Schiessel et al.
Saito, 2014 [64]	-T1–4 -≤5 cm from AV	-EAS/LAM infiltration -Fecal incontinence
Shirouzu, 2017 [70] (Review)	-T1–3 -1–5 cm AV -Well-moderately diff. adenoca.	-T4 -Fixed tumors -EAS/LAM infiltration -Untreatable distant metastases -Poorly diff. adenoca. -Poor anal function -Severe preoperative pathologies (cardiac failure, liver cirrhosis, renal dysfunction, respiratory dysfunction) -Psychiatric disease
Park, 2019 [56]	-Tumor’s response to nCRT on restaging MRI -Evaluation of ymrT stage and ymrCRM status	-Poor nCRT responders
Piozzi, 2021 [57]	-≤4 cm from AV -After nCRT for cT3-T4 -(y)cT4 if curative resection is technically feasible at the pre-operative MRI -Conversion from an ultra-low AR in case of involvement/threatening of the distal gross margin in the resected specimen or in case of stapler failure for any reason	-EAS/LAM infiltration (at restaging MRI after nCRT) -Abundant mucinous component -Anal canal involvement below DL (requiring total ISR) -Fecal incontinence -Patient’s refusal

AV: Anal verge; DL: Dentate line; DRE: Digital rectal examination; DRM: Distal resection margin; EAS: External anal sphincter; ECOG PS: Eastern Cooperative Oncology Group scale of Performance Status; EUS: Endoscopic ultrasound; IAS: Internal anal sphincter; ISR: Intersphincteric resection; LAM: levator ani muscle; LRC: Low rectal cancer; MRI: Magnetic resonance imaging; nCRT: Neoadjuvant chemoradiotherapy; RT: Radiotherapy.

**Table 2:** Characteristics of patients and tumors from published series on ISR.

First Author, Year	Country	*n*	Age	Sex, M	Distance-AV, cm	nCRT	Approach	ISR	Type (Par, Subt, Tot, ESR)	cT Stage	Stage 0/I/II/III/IV	DRM, cm	CRM, mm	R0	FU, mo	LR Rate	DM	CT	OS-5 Years	DFS-5 Years	LRFS-5 Years
Kohler [43], 2000	Germany	31	60	55%	1.3 ± 0.9 (DL)	0%	O	T-P	0/31/0/0	T1–3	0/18/4/9/0	1.6 ± 0.8	nr	100%	6.8 ± 3.7 (y)	9.7%	19.4%	35%	79%	nr	nr
Vorobiev [58], 2004	Russia	27	55 (26–75)	59%	1.0 (0.5–1.5) (DL)	7%	O	P	0/0/27/0	T2–3	0/8/18/1/0	1.9 (1.5–2.6)	0.8 (0.6–1.5)	100%	38 (14–66)	0	11.1%	3.7%	92.5% (3-y)	88.9% (3-y)	0%
Schiessel [19], 2005	Austria	121	65.2	68%	3 (1–5) (AM)	nr	O	T-P	nr	T1–3	DK. A49, B33, C37	nr	nr	nr	94 (24–185)	5.3%	nr	nr	nr	nr	nr
Rullier [45], 2005	France	92	65 (25–86)	62%	3 (1.5–4.5)	88%	O/L	T-P	nr	T1–4	nr	2 (0.5–3)	5 (0–15)	89%	40 (63%)	2% (63%)	19% (63%)	nr	81%	70%	nr
Hohenberger [46], 2006	Germany	65	nr	nr	<2 (DL)	54%	O	T-P	60/0/0/0	nr	nr	1.5–2.0	nr	nr	nr	22.7%	nr	nr	85.1%	nr	nr
Chamlou [48], 2007	France	90	59 (27–82)	65%	3.5 (2.2–5.2)	41%	O	T-P	63/27/0/0	T1–4	0/37/16/25/5	1.2 (0.5–3.5)	nr	94.4%	56.2 (13.3–168.4)	8.8%	8.8%	nr	82%	75%	nr
Portier [49], 2007	France	173	64 ± 11	33%	4.1 ± 1.4	53%	O	T-P	173/0/0/0	T1–4	0/74/46/53/0	2.6 ± 1.2	nr	96%	66.8 ± 52.1	10.6%	nr	nr	86.1%	83.9%	nr
Akasu [50], 2007	Japan	106	55 (26–75)	78%	3 (1–5)	0%	O/L	T-P	90/0/16/6	T1–3	0/45/20/38/3	1.2 (0.3–4)	nr	97%	3.5 (0.9–11.7) (y)	5.7%	10%	18%	91%	82%	88%
Krand [59], 2009	Turkey	47	57 (27–72)	66%	3.3 (1.5–5)	100%	O	P	47/0/0/0	T2–3	0/nr/nr/25/0	1.2 ± 0.3	5 ± 2.3	98%	67.5 (9–132)	2.1%	15.2%	53.2%	85%	82%	nr
Han [60], 2009	China	40	62 (34–73)	60%	1.5 (0.5–5.0) (DL)	2.5%	O	P	23/0/5/0	T1–2	0/18/6/16/0	2.1 (2–5)	nr	100%	43 (12–94)	5%	2.5%	7.5%	97%	86%	nr
Weiser [61], 2009	U.S.A.	44	54 (28–78)	57%	5 (3–6)	100%	O	P	nr	T1–3	11/16/12/5/0	nr	nr	92%	47 (33–59)	0%	16%	nr	nr	96%	83%
Kuo [62], 2011	Taiwan	26	51 (26–71)	61%	3.5 (2.5–5)	88%	O	P	26/0/0/0	T1–3	0/14/2/9/0	1.4 (0.1–4.5)	11 (1–31)	87%	55 (8–93)	7.7%	15%	nr	83%	76%	nr
Gong [63], 2012	China	43	53	63%	<5	0%	O	P	nr	T1–2	nr	nr	nr	100%	20 (12–42)	0%	0%	nr	nr	nr	nr
Zhang [51], 2013	China	60	55 (30–77)	65%	4.2 (3–5)	30%	O	T-P	34/0/26/0	T1–4	0/28/21/11/0	1.9 (1.0–3.2)	≥1 mm	100%	49 (18–90)	10%	6.6%	nr	90%	83.3%	nr
Tokoro [52], 2013	Japan	30	60 ± 10	40%	0.9 ± 0.8 (DL)	0%	O	T-P	12/4/14/4	Tis–T3	1/16/5/7/1	0.7 (0.3–2.2)	3 (0.5–9)	93% (CRM)	56.2 (13.3–168.4)	20%	16%	nr	76.5%	68.4%	nr
Akagi [53], 2013	Japan	124	65 (32–81)	62%	3 (1–4)	0%	O	T-P	nr	T1–3	0/43/41/40/0	nr	nr	97.6% (CRM)	85 (14–122)	4.8%	10.5%	46.8%	84.2–78.6%	90.5–83.6%	81.7%
Saito [64], 2014	Japan	199	59 (27–80)	72%	3.8	25%	O	P	64/80/55/41	T1–4	9/69/46/75/0	nr	19.6% (≤1 mm)	80.4%	78 (12–164)	13.6%	nr	48%	78.3% (7-y)	66.7% (7-y)	80.3% (7-y)
Abdel-Gawad [65], 2014	Egypt	55	nr	nr	2.3 (0–5)	45%	O/L	T-P	35/0/20/20	T1–3	nr	nr	nr	94.5% (CRM)	1.5 (1–4.6) (y)	5.4%	12.7%	nr	88.7% (3-y)	82.6% (3-y)	85.2% (3-y)
Koyama [88], 2014	Japan	77	63 (24–86)	73%	nr	9%	nr	nr	nr	T1–4	0/20/25/32/0	nr	nr	nr	69 (56–87)	7.8%	nr	nr	76.4%	nr	93.5%
Mahalingam [54], 2017	India	33	50 (26–69)	64%	3 (1.5–5)	91%	nr	T-P	nr	nr	nr	2 (0.4–4)	nr	100%	48 (18–83)	0%	5%	nr	95% (3-y)	nr	nr
Klose [89], 2017	Germany	60	67 (41–86)	72%	3.4 (1–5)	73%	nr	nr	nr	T1–4	0/36/12/9/3	nr	nr	95%	58 (11–210)	nr	nr	23%	80%	69%	nr
Matsunaga [55], 2019	Japan	197	61 (33–80)	70%	4 (0.6–6.5)	0%	nr	T-P	88/62/47/0	T1–3	nr	nr	0.3 (0.01–2)	88% (CRM)	68 (9–182)	nr	nr	nr	88.3%	76.9%	nr
Molnar [90], 2019	Romania	37	66 ± 11	65%	10–40	nr	nr	nr	nr	nr	0/7/13/16/1	nr	nr	87% (CRM)	62 (55–80)	5.4%	5.4%	nr	71%	nr	nr
Park [56], 2019	Korea	147	61 ± 11	72%	2.8 ± 1.0	100%	L/R	T-P	31/95/21/0	T2–4	33/36/42/36/0	nr	nr	95%	34 (8–94)	11.6%	22.4%	nr	nr	64.9% (3-y)	nr
Kim [68], 2021	Korea	590	58 ± 11	59%	3.3 ± 1.9	47%	R	TA	155/93/42/70	Tis–T3	41/103/59/77	1.5 ± 1.3	8 ± 6	DRM ≤ 10 mm (45.4%), CRM ≤ 1 mm (7.8%)	43 (21–59)	2.4% (PS)	15.1% (PS)	nr	90.8% (PS)	81.6% (PS)	nr
Piozzi [57], 2021	Korea	161	59 (51–68)	75%	3 (2.5–3.5)	71%	L/R	T-P	nr	T1-4	15/51/34/44/17	0.8 (0.5–1.5)	0.3 (0.2–0.5)	91.3% (CRM)	55 (34.5–77.5)	11.1%	26.1%	55.9%	80%	64%	87%

AM: Anal margin; AV: Anal verge; CRM: Circumferential resection margin; CT: Adjuvant chemotherapy; DFS: Disease-free survival; DK: Dukes stage; DL: Dentate line; DM: Distant metastases; DRM: Distal resection margin; ESR: External sphincter resection; FU: Follow-up; ISR: Intersphincteric resection; L: Laparoscopic; O: Open; OS: Overall survival; LR: Local recurrence rate; LRFS: Local recurrence free survival; M: Male; nCRT: Neoadjuvant chemoradiotherapy; P: Perineal only; PS: Propensity score matched group; R: Robotic; TA: Totally transabdominal; T-P: Transabdominal and perineal.

## Abbreviations

AB	Anterior bundle of the longitudinal muscle
AC	Anal canal
ACL	Anococcygeal ligament
AM	Anal margin
APR	Abdominoperineal resection
AV	Anal verge
BM	Bulbospongiosus muscle
CAA	Coloanal anastomosis
CEA	Carcinoembryonic antigen
CI	Confidence interval
CM	Circumferential muscle
CRM	Circumferential margin
CSP	Corpus spongiosum of the penis
CT	Adjuvant chemotherapy
Cx	Coccyx
DFS	Disease free survival
DK	Dukes stage
DL	Dentate line
DM	Distant metastases
DRE	Digital rectal examination
DRM	Distal resection margin
EAS	External anal sphincter
ECOG PS	Eastern Cooperative Oncology Group scale of Performance Status
ESR	External sphincter resection
EUS	Endoscopic ultrasound
FU	Follow-up
HL	Hiatal ligament
HR	Hazard ratio
JSCCR	Japanese Society for Cancer of the Colon and Rectum
IAS	Internal anal sphincter
ISG	Intersphincteric groove
ISP	intersphincteric plane
ISR	Intersphincteric resection
LAM	Levator ani muscle
LISR	Laparoscopic intersphincteric resection
LM	Longitudinal muscle
LN	Lymph node
LR	Local recurrence
LRC	Low rectal cancer
LRFS	Local recurrence free survival
MRI	Magnetic resonance imaging
nCRT	Neoadjuvant chemoradiotherapy
OISR	Open intersphincteric resection
OR	Odds ratio
OS	Overall survival
PL	Parks’ ligament
PR	Prostate
RFS	Relapse free survival
RIP	Raphe of the iliococcygeus and pubococcygeus muscle
RISR	Robotic intersphincteric resection
RS	Urethral rhabdosphincter
RT	Radiotherapy
RU	Rectourethralis muscle
RVS	Rectovaginal septum
SP	Single port
TILME	Total intersphincteric longitudinal muscle excision
TME	Total mesorectal excision
UR	Urethra
VM	Vaginal smooth muscle layer
AB	Anterior bundle of the longitudinal muscle
AC	Anal canal
ACL	Anococcygeal ligament
AM	Anal margin
APR	Abdominoperineal resection
